# Moxd1 Is a Marker for Sexual Dimorphism in the Medial Preoptic Area, Bed Nucleus of the Stria Terminalis and Medial Amygdala

**DOI:** 10.3389/fnana.2017.00026

**Published:** 2017-03-27

**Authors:** Yousuke Tsuneoka, Shinji Tsukahara, Sachine Yoshida, Kenkichi Takase, Satoko Oda, Masaru Kuroda, Hiromasa Funato

**Affiliations:** ^1^Department of Anatomy, Faculty of Medicine, Toho UniversityTokyo, Japan; ^2^Division of Life Science, Graduate School of Science and Engineering, Saitama UniversitySaitama, Japan; ^3^Precursory Research for Embryonic Science and Technology (PRESTO), Japan Science and Technology AgencySaitama, Japan; ^4^Laboratory of Psychology, Jichi Medical UniversityTochigi, Japan; ^5^International Institutes for Integrative Sleep Medicine (WPI-IIIS), University of TsukubaIbaraki, Japan

**Keywords:** *Moxd1*, calbindin, masculinization, SDN, MPOA, BNST, amygdala, molecular marker

## Abstract

The brain shows various sex differences in its structures. Various mammalian species exhibit sex differences in the sexually dimorphic nucleus of the preoptic area (SDN-POA) and parts of the extended amygdala such as the principal nucleus of the bed nucleus of the stria terminalis (BNSTpr) and posterodorsal part of the medial amygdala (MePD). The SDN-POA and BNSTpr are male-biased sexually dimorphic nuclei, and characterized by the expression of calbindin D-28K (calbindin 1). However, calbindin-immunoreactive cells are not restricted to the SDN-POA, but widely distributed outside of the SDN-POA. To find genes that are more specific to sexually dimorphic nuclei, we selected candidate genes by searching the Allen brain atlas and examined the detailed expressions of the candidate genes using *in situ* hybridization. We found that the strong expression of *monooxygenase DBH-like 1* (*Moxd1*) was restricted to the SDN-POA, BNSTpr and MePD. The numbers of *Moxd1*-positive cells in the SDN-POA, BNSTpr and MePD in male mice were larger than those in female mice. Most of the *Moxd1*-positive cells in the SDN-POA and BNSTpr expressed calbindin. Neonatal castration of male mice reduced the number of *Moxd1*-positive cells in the SDN-POA, whereas gonadectomy in adulthood did not change the expression of the *Moxd1* gene in the SDN-POA in both sexes. These results suggest that the *Moxd1* gene is a suitable marker for sexual dimorphic nuclei in the POA, BNST and amygdala, which enables us to manipulate sexually dimorphic neurons to examine their roles in sex-biased physiology and behaviors.

## Introduction

Sex differences in brain structures underlie behavioral or physiological sex differences such as sexual behaviors, parenting and aggression. In rodents, structural sex differences have been well documented in the medial preoptic area (MPOA), bed nucleus of the stria terminalis (BNST) and posterodorsal part of the medial amygdala (MePD; de Vries and Södersten, 2009; Semaan and Kauffman, 2010; Campi et al., 2013), which regulate male sexual behaviors (Hull and Dominguez, 2007), female sexual receptivity (Veening et al., 2014) and paternal and maternal behaviors (Numan, 2007).

The MPOA exhibits sex differences in terms of cell number, spine density, fiber density and volume of the subregion (Gorski et al., 1978; Simerly et al., 1984; de Vries and Södersten, 2009). Among the MPOA subregions, the sexually dimorphic nucleus of the preoptic area (SDN-POA) exhibits the most apparent difference between sexes. The volume of the SDN-POA and the number of neurons in the SDN-POA of male rats are greater than those of female rats (Gorski et al., 1978). Similar sex differences were reported in the MPOA of a variety of mammals including hamster, gerbil, California mouse, ferret, guinea pig and human (Campi et al., 2013). The sex differences in the SDN-POA is not affected by testosterone after sexual maturation but is significantly affected by testosterone during the perinatal period (Gorski et al., 1978).

High testosterone levels during the perinatal period induces the masculinization of the brain, a permanent characteristics toward male reproductive behaviors, as well as sexually dimorphic changes in brain structure (Becker et al., 2005; de Vries and Södersten, 2009; Semaan and Kauffman, 2010). Despite the close relationship, it remains to be elucidated whether the sexual dimorphism in the SDN-POA plays a major role in sex-biased or sex-specific functions and behaviors. Large lesions of the MPOA including the SDN-POA caused an apparent deficiency in male sexual behavior in sexually experienced rats (Heimer and Larsson, 1967; Ginton and Merari, 1977), while small lesions of the SDN-POA did not induce significant deficiency in male sexual behaviors in rats and ferrets (Arendash and Gorski, 1983; Turkenburg et al., 1988; Cherry and Baum, 1990). It is noted that discrete bilateral lesions of the SDN-POA delay the onset and decrease sexual behaviors in sexually naive adult male rats (De Jonge et al., 1989).

Moreover, there have been inconsistent reports on the relationship between the volume of the SDN-POA and male sexual behaviors. Prenatally stressed males exhibited a reduced volume of the SDN-POA as well as male sexual activities (Anderson et al., 1986). In contrast, a prenatal testosterone treatment in female rats increased the volume of the SDN-POA but did not induce male-like sexual behaviors (Ito et al., 1986). The inhibition of aromatase during the perinatal period in male rats reduced the SDN-POA volume, but did not affect male sexual behaviors (Brand et al., 1991). To address whether the SDN-POA is involved in reproductive behaviors, optogenetic and pharmacogenetic manipulations of the SDN-POA could be necessary using a molecular marker restricted to the SDN-POA.

Calbindin D-28K (*calbindin 1*) has been used to visualize the SDN-POA of rats and mice (Sickel and McCarthy, 2000; Edelmann et al., 2007; Bodo and Rissman, 2008). The cluster of calbindin-immunoreactive (ir) cells in the MPOA also shows sex differences and responsiveness to perinatal hormones (Bodo and Rissman, 2008; Orikasa and Sakuma, 2010). Additionally, sex differences in calbindin-ir cells of the MPOA have been demonstrated in musk shrews and common marmosets (Moe et al., 2016a,b).

Similar to the SDN-POA, the volume and cell number of the MePD and the principal nucleus of the BNST (BNSTpr) of male mice are larger than those of female mice (Hisasue et al., 2010; Campi et al., 2013). The perinatal testosterone level determines the volume and cell number of the BNSTpr (Hisasue et al., 2010). Then MePD and BNSTpr belong to the extended amygdala which is involved in male sexual behavior (Newman, 1999). BNSTpr neurons are also calbindin-positive and the number of calbindin-ir cells in the BNSTpr has been shown to be male-biased (Gilmore et al., 2012; Moe et al., 2016b). When female mice are injected with estradiol in the postnatal period, the number of calbindin-ir cells in the BNSTpr is increased and similar to that of male mice in adulthood (Gilmore et al., 2012).

Although calbindin is the most commonly used marker for the sexually dimorphic nuclei, calbindin is not restricted to the sexually dimorphic nuclei but broadly distributed around the sexually dimorphic nuclei, which makes it difficult to manipulate neurons of the sexually dimorphic nuclei using the calbindin gene for optogenetic and pharmacogenetic approaches. This prompted us to look for a better gene marker for sexually dimorphic nuclei. In the current study, we searched candidate genes that are highly expressed in the SDN-POA using the Allen brain atlas and subsequent *in situ* hybridization (ISH). We found that the strong expression of *monooxyganase DBH-like1* (*Moxd1*) was restricted to the SDN-POA, BNSTpr and MePD in a male-biased manner. To the best of our knowledge, this is the first report of *Moxd1* expression in the SDN-POA, BNSTpr and MePD. Based on a male-biased expression, we further hypothesized that the perinatal hormonal milieu affects the *Moxd1* expression. In fact, neonatal castration reduced the number of *Moxd1*-positive cells in the SDN-POA, BNSTpr and MePD.

MOXD1 belongs to the copper-dependent monooxygenase family (Xin et al., 2004). However, the substrate of MOXD1 has not been identified and therefore the functional role of MOXD1 in neurons remains unknown. The current findings suggest that *Moxd1* is a useful maker for the sexually dimorphic nuclei and may be involved in the regulation of sex-biased physiology and behaviors.

## Materials and Methods

### Animals

Breeding pairs of C57BL/6J mice (RRID:IMSR_JAX:000664) were obtained from Japan SLC Inc. and CLEA Japan to build and maintain our breeding colony. The breeding colony was periodically refreshed with new breeding pairs. Mice were raised under controlled conditions (12 h light/dark cycle, lights on at 8:00 AM, 23 ± 2°C; 55 ± 5% humidity, and *ad libitum* access to water and food). Mice were weaned at 4 weeks of age and housed in groups of four or five.

For assessing *Moxd1* expression in the MPOA, five groups of mice were used: intact male, castrated male, neonatally-castrated male, intact female and ovariectomized female. Eight intact male and female mice were sacrificed and their brains were sampled as described below. Another six adult male mice were castrated, and 2 weeks later, their brains were sampled. Additionally, six ovariectomized female mice were sampled in the same manner. Six infant male mice were castrated on the day of birth and raised under normal conditions (Becker et al., 2005). Four mice from the intact male, neonatally-castrated male and intact female groups were also used for assessing *Moxd1* expression in the BNSTpr and MePD. The same four intact male mice and female mice were used to confirm no sexual difference in the other areas. In addition, the same four males were used for assessing colocalization of *Moxd1* and calbindin D. All mice were 10–20 weeks old when sampled. All procedures were carried out in accordance with the Guidelines for Animal Experiments of Toho University. All animal experimentation was approved by the Institutional Animal Care and Use Committee of Toho University (Approved protocol ID #15-52-254).

### Sample Preparation

Mice were deeply anesthetized with sodium pentobarbital (50 mg/kg, i.p.) and then perfused transcardially with 4% (w/v) paraformaldehyde (PFA) in 0.01 M phosphate-buffered saline (PBS, pH 7.4). The brains were removed, postfixed in 4%PFA/PBS at 4°C overnight, followed by cryoprotection in 30% (w/v) sucrose in PBS for 2 days, embedded in Surgipath (FSC22, Leica Biosystems), and then stored at −80°C until cryosectioning. To assess the expression of genes of interest in the MPOA, 40 μm-thick serial coronal sections were prepared to cover the entire MPOA according to the mouse brain atlas (Franklin and Paxinos, 2007) and stored in a cyoprotectant solution (30% glycerol, 30% ethylene glycol, 0.05 M phosphate buffer) at −30°C until use. We used a set of every third sections from the serial sections to examine the expression of target molecules.

### Database Search for a Marker Gene

Candidate genes for an area marker of the SDN-POA were acquired from the Allen Gene Expression Atlas in the Allen Brain Atlas database^1^. To investigate specific gene expressions in the MPOA, we used the following parameters. The coordinates of the region of interest were as follows: AP: 5.000–5.800 mm, DV: 5.000–6.600 mm, L: 5.800–6.200 mm. This coordinates contains the whole MPOA and a part of the BNST. Other parameters were not changed from default values. The search provided more than 500 candidate genes in descending order of fold-change together with ISH images. The authors visually examined the expressions of all candidate genes using the Allen brain atlas to find any genes that were strongly and specifically expressed in the MPOA. Our *in silico* search for MPOA marker genes selected nine genes that were localized to a specific subregion in the MPOA. We performed ISH of these genes in our mouse samples and confirmed the expression of the *Moxd1* gene in the subregions of the MPOA.

### ISH

One antisense and one sense riboprobes were used to assess the expression of *Moxd1* mRNA (RefSeq ID: NM_021509.5, 422-2743 bp). cDNA fragments were amplified with polymerase chain reaction, inserted into the multi cloning site of a pGEM-T plasmid (A3600, Promega), and transformed into DH5α *E. coli*. The cDNA was verified by sequencing (Hokkaido system science, Japan). The plasmid DNA was extracted and purified after liquid culture, and the template cDNA was produced using polymerase chain reaction using the specific primers (5′-ATTTAGGTGACACTATAG-3′) and (5′-TAATACGACTCACTATAGGG-3′). Probes were transcribed by SP6 RNA polymerase (P1085, Promega) for antisense probe or T7 RNA polymerase (10881767001, Roche) for sense probe in the presence of digoxigenin-labeled UTP (Dig labeling mix; Roche Diagnostics, Basel, Switzerland) followed by precipitation with LiCl with ethanol.

Brain sections were processed for ISH as previously described (Tsuneoka et al., 2013, 2015) with some modifications. Briefly, the sections were washed with PBS containing 0.1% Tween-20 (PBT), postfixed with 4% PFA in PBS for 10 min at room temperature. Then, the sections were immersed in methanol containing 0.3% H_2_O_2_ for 10 min, followed by acetylation with 0.25% acetic anhydride in 0.1 M triethanolamine (pH 8.0). The hybridization solution contained 50% deionized formamide, 5× standard saline citrate (SSC, pH 7.0), 5 mM ethylene-diaminetetraacetic acid (pH 8.0), 0.2 mg/ml of yeast tRNA, 0.2% Tween-20, 0.2% sodium dodecyl sulfate, 10% dextran sulfate, and 0.1 mg/ml of heparin. The sections were prehybridized at 58°C in the mixture of the hybridization solution and PBT (1:1) for 30 min, immersed in the hybridization solution for 15 min, and then hybridized with the riboprobes (1 μg/ml) at 58°C for 16 h. After hybridization, the sections were washed twice with 2× SSC containing 50% deionized formamide at 58°C for 10 min, incubated with RNase A solution (20 μg/ml, R4642, Sigma) and avidin (0.1 μg/ml, 013-21013, Wako pure chemicals, Japan) at 37°C for 60 min, and rinsed twice in 2× SSC and four times in 0.2× SSC at 37°C (15 min each).

To visualize digoxigenin-labeled riboprobe, the sections were incubated in a peroxidase-conjugated anti-digoxigenin antiserum (1:1000, Roche Diagnostics, RRID:AB_514499) with biotin (0.5 μg/ml, 021-08712, Wako pure chemicals). After a 2-h incubation in the antibody solution at room temperature, the sections were washed and immersed in 0.1 M boric buffer (pH 8.5) containing 4 μM biotin-labeled tyramide, 4% dextran sulfate, 0.05 mg/ml iodophenol, and 0.003% H_2_O_2_ for 30 min. They were immersed in a cocktail of Alexa 647-conjugated streptavidin (1:10,000, Life Technologies, Grand Island, NY, USA) and Hoechst 33342 (1 μg/ml, Life Technologies). The sections were mounted on a glass slide with Gel/Mount. The sections were washed three times with PBS.

For double ISH for *Moxd1* and *Trh*, an antisense riboprobe for *Trh* mRNA (RefSeq ID: NM_009426.3, 88–990 bp) was synthesized using SP6 RNA polymerase (P1085, Promega) in the presence of dinitrophenol-labeled UTP (NEL555001EA, PerkinElmer, Waltham, MA, USA). Double ISH was performed using hybridization solution containing both digoxigenin-labeled riboprobe for *Moxd1* and dinitrophenol-labeled riboprobe for *Trh* mRNA (1 μg/ml for each). Digoxigenin-labeled *Moxd1* riboprobe was visualized as described above. Dinitrophenol-labeled *Trh* riboprobe was visualized using a peroxidase-conjugated anti-dinitrophenyl antibody (1:2500, FP1129, PerkinElmer, RRID:AB_2629439), followed by the incubation in 0.1 M boric buffer (pH 8.5) containing 4 μM Alexa 568-labeled tyramide, 4% dextran sulfate, 0.05 mg/ml iodophenol and 0.003% H_2_O_2_ for 30 min.

### ISH Combined with IHC

To visualize *Moxd1* mRNA and calbindin D, *Moxd1-*ISH performed sections were further processed for the immunostaining for calbindin D. Similarly, to visualize *Moxd1* mRNA, *Trh* mRNA and oxytocin, sections that were performed for double ISH for *Moxd1* and *Trh* mRNAs were further processed for the immunostaining for oxytocin-neurophysin I. To visualize *Trh* mRNA, calbindin D and oxytocin, *Trh*-ISH performed sections were processed for double immunostaining for calbindin D and oxytocin.

For immunostaining, sections were incubated in a mouse anti-calbindin D-28K antibody (1:1000, C9848, Sigma Aldrich, RRID:AB_476894) or in the cocktail of mouse anti-calbindin and goat anti-neurophysin I antibodies (1:2000, sc-7810, Santa Cruz, RRID:AB_650167) overnight. Then, the sections were washed, immersed in a cocktail of Alexa 568-conjugated anti-mouse IgG antibody (1:250, Thermoscientific, RRID:AB_2534013) and/or Alexa 488-conjugated anti-goat IgG antibody (1:500, Jackson Immunoresearch, RRID:AB_2340428). Immunostained sections were mounted on a glass slide with Gel/Mount.

### Histological Analysis

**Figure 1:**
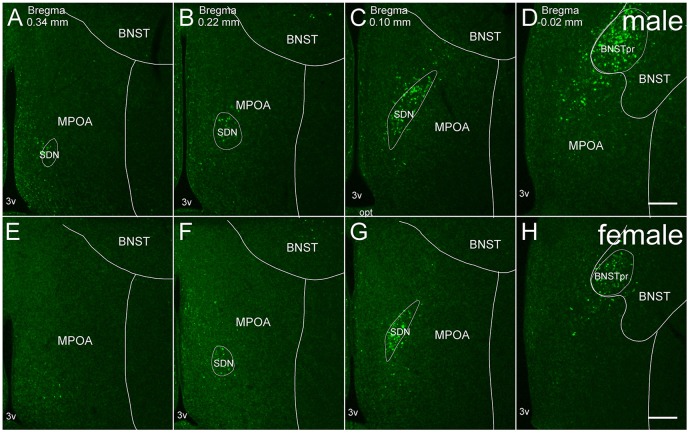
**Representative fluorescent images of *in situ* hybridization for *Moxd1* mRNA in the MPOA and adjacent areas. (A–D)** Coronal sections of male mice along the anterior-posterior axis. **(E–H)** Coronal sections of female mice along the anterior-posterior axis. BNSTpr, principal nucleus of the bed nucleus of the stria terminalis; MPOA, medial preoptic area; SDN, sexually dimorphic nucleus of the preoptic area; 3v, third ventricle. Scale bars: 200 μm.

To count *Moxd1*-positive cells in the SDN-POA, we used a set of three coronal sections 120 μm apart. To fix the position of the three sections, the most rostral section of the three sections was set to the level where the rostral end of the commissural part of the anterior commissure was located (approximately Bregma +0.22 mm). Thus, Figures 1B–D indicate the typical three sections that we used for cell counting. Each set of three sections from intact, castrated, or neonatally castrated males, or intact and ovariectomized females was used for single ISH and then processed for positive cell counting.

To evaluate the colocalization of *Moxd1* and calbindin, four sets of MPOA sections from different male mice were randomly selected and then *Moxd1*- and calbindin-positive cells were counted and averaged in the SDN-POA, the border of which was determined by the distribution of the *Moxd1* signals. For the other areas, every third coronal section from the whole brain except the olfactory bulb from different male mice (*n* = 4) were randomly selected and stained for *Moxd1* ISH combined with IHC for calbindin. Identification of the brain areas was done according to the chemoarchitectonic atlases (Dong, 2008; Paxinos and Watson, 2010; Martínez-García et al., 2012) and our previous study (Tsuneoka et al., 2013, 2015).

Fluorescent microscopic images of brain sections were taken using a Nikon microscope Eclipse Ni under a 20× objective equipped with a confocal detection system A1R (Nikon Instruments Inc., Tokyo, Japan). To obtain fluorescent images, each channel was collected separately with single-wavelength excitation and then merged to produce a composite image. Each image was obtained as five-layer *z*-stack images, and the optical thickness of the sections was 1.0 μm. Experimental controls were prepared in which the primary antibodies or riboprobes were omitted from the reaction solution to confirm no detectable signal. For ISH-stained sections, some non-specific granule-like signals were observed but easily distinguished from cytoplasmic-specific staining. In addition, specific staining of antisense probes was confirmed by the staining using sense probes (data not shown).

Images were analyzed using ImageJ software (version 1.50i, NIH, USA, RRID:SCR_003070). Each image was binarized by the fixed threshold value and the cell was marked manually on the threshold image. Threshold was determined to be above background or nonspecific signals on the control sections, and the same threshold was used through analysis in all samples. Since all procedures of brain sampling, ISH and IHC were performed in an exactly same time course under controlled temperature, the fixed threshold work to evaluate positive cells of different mice. Cell counting was conducted in three sections containing the SDN-POA unilaterally, and the summed value from the three sections was applied to a statistical analysis. In the other brain regions, cell counting was conducted in one section which was selected to maximize counting area.

To calculate the proportion of double labeling, we separately marked singly labeled cells and doubly labeled cells of *Moxd1* mRNA and calbindin on the threshold image. The double-labeling was judged when the signals were observed just around cell nuclei and overlapped each other.

To quantify the sex difference of the BNSTpr and MePD, ISH signal-positive area of the threshold image was measured instead of cell counting because the cell density was too high to separate each cell signals. All histological procedures were done without the observer knowing the sex of the samples.

### Statistical Analysis

Data were analyzed using Welch’s *t*-test or Welch’s one-way analysis of variance (ANOVA) followed by *post hoc* comparisons using Welch’s *t*-test to address unequal variances among groups if the data match the statistical premise of the tests. The *P* values of all multiple comparisons were adjusted appropriately using the Holm’s method, also called a sequential Bonferroni (Sokal and Rohlf, 1995). An adjusted *P* < 0.05 was regarded as statistically significant. All statistical analyses were conducted using software R 2. 14. 0. Quantitative data were presented as the mean ± SEM.

## Results

### *Moxd1* Expression in the SDN-POA

*Moxd1* mRNA-positive cells observed in and around the MPOA were restricted to the SDN-POA and BNSTpr of adult mice. *Moxd1* mRNA-positive cells were distributed in the entire SDN-POA and narrowly continuous with the BNSTpr (Figure 1). On the other hand, *Moxd1* mRNA-positive cells were only a few in the remaining part of the MPOA.

**Figure 2:**
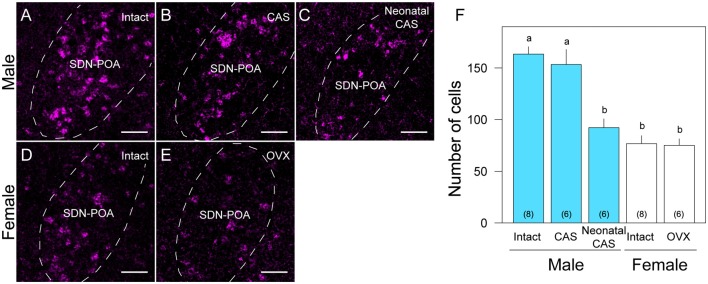
**Sex and gonadal steroid dependent differences in *Moxd1*-positive cells in the SDN-POA.** Representative images of intact male **(A)**, castrated (CAS) male **(B)**, neonatally castrated male **(C)**, intact female **(D)** and ovariectomized (OVX) female **(E)** mice. Dashed lines indicate the boundaries of the SDN-POA. Scale bars: 50 μm. **(F)** Difference in the number of *Moxd1*-positive cells. Significant differences were found between experimental groups indicated by a and b. The number in parenthesis of each bar indicates the number of mice. (*P* < 0.05, Welch’s *t*-test with Holm’s multiple correlation).

Overall, the number of *Moxd1*-positive cells in the SDN-POA were significantly affected by sex and gonadectomy (*F*_(4,13.3)_ = 25.4, *P* < 0.001), although their distributions in the MPOA including the SDN-POA were not different (Figures 1, 2). The number of *Moxd1*-positive cells in the SDN-POA of male mice was significantly larger than that of female mice (*Post hoc* test, *P* < 0.001, Figures 1, 2A,D,F). To examine the effect of castration on the number of *Moxd1*-mRNA positive cells in the SDN-POA of male mice (Becker et al., 2005), we performed castration in the adult or neonatal period. Although the distributions of *Moxd1* mRNA-positive cells of the castrated and neonatally castrated mice were similar to that of intact adult mice, the number of *Moxd1* mRNA-positive cells of neonatally castrated mice was significantly smaller than those of castrated mice and intact mice (*P* < 0.01 for both) and similar to that of female mice (*P* = 0.544, Figure 2F). The number of *Moxd1* mRNA-positive cells of intact males was similar to that of castrated males (*P* = 0.870). Additionally, we examined the effects of an ovariectomy in adult females on the number of *Moxd1* mRNA-positive cells in the SDN-POA and found that the number was not significantly different between intact and ovariectomized female mice (*P* = 0.621; Figures 2D–F).

### Colocalization of *Moxd1* and Calbindin in the SDN-POA

**Figure 3:**
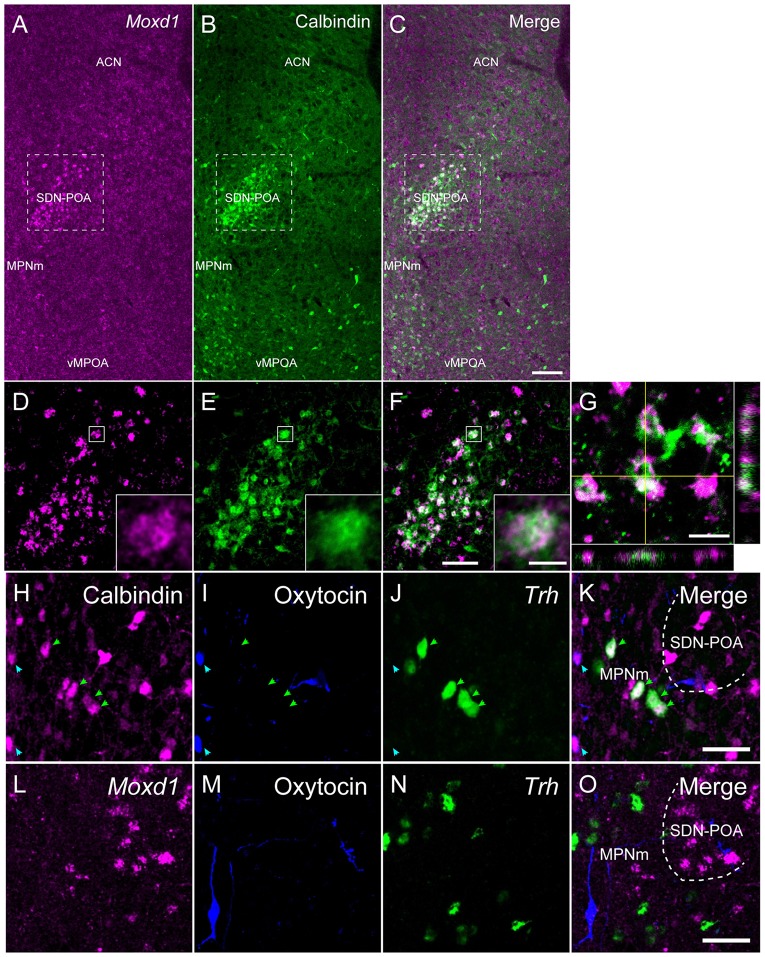
**Comparison of the distribution of *Moxd1*-positive cells with calbindin-immunoreactive cells in the MPOA of male mice. (A–C)** Representative images of *Moxd1* (magenta) and calbindin (green) at low magnification. Scale bar: 100 μm. **(D–F)** High magnification images indicated by rectangles in **(A–C)**. Scale bar: 50 μm. The inset shows a high magnification view of a double-labeled cell indicated by small rectangle. Scale bar: 5 μm. **(G)** Orthogonal view of *Moxd1-* and calbindin-positive cells. Sacle bar: 10 μm. ACN, anterior commissural nucleus; MPNm, medial preoptic nucleus, medial part; vMPOA, ventral part of the MPOA. **(H–K)** Representative images of calbindin (magenta), oxytocin-neurophysin I (blue) and *Trh* (green). Arrowhead indicates colocalization of calbindin and oxytocin (blue), or calbindin and *Trh* (magenta). **(L–O)** Representative images of *Moxd1* (magenta), oxytocin-neurophysin I (blue) and *Trh* (green). Scale bars: 20 μm.

As previously reported (Orikasa and Sakuma, 2010), calbindin-ir cells were aggregated in the SDN-POA and broadly distributed in the MPOA (Figure 3). Double labeling of *Moxd1* mRNA and calbindin protein confirmed that a large number of the *Moxd1* mRNA-positive cells expressed calbindin (Figures 3A–G; *n* = 4, 70.4 ± 6.1%). In the SDN-POA, the population of *Moxd1* mRNA-positive cells in calbindin-ir cells was relatively small (*n* = 4, 57.8 ± 2.6%).

Calbindin-ir and *Moxd1* mRNA-negative cells were also found in the MPN outside of the SDN-POA, the ventral part of the MPOA (vMPOA) and the anterior commissural nucleus (ACN; Figure 3B). In contrast, the *Moxd1* mRNA-positive and calbindin-negative cells were found only in the SDN-POA (Figure 3A). Most of oxytocin-neurophysin I-ir cells, which were localized mainly in the ACN and periventricular preoptic area (PVPOA), were immunopositive for calbindin and negative for *Moxd1* (Figures 3H–K). Most of *Trh*-expressing cells, which were localized mainly in the ACN, MPN and vMPOA, were immunopositive for calbindin and negative for *Moxd1* mRNA-positive cells (Figures 3K,O). No *Moxd1* positive signal was found in oxytocin-neurophysin I-positive cells or *Trh* mRNA-positive cells in the MPOA (Figures 3L–O).

### Distribution of *Moxd1* mRNA in Various Brain Regions

**Figure 4:**
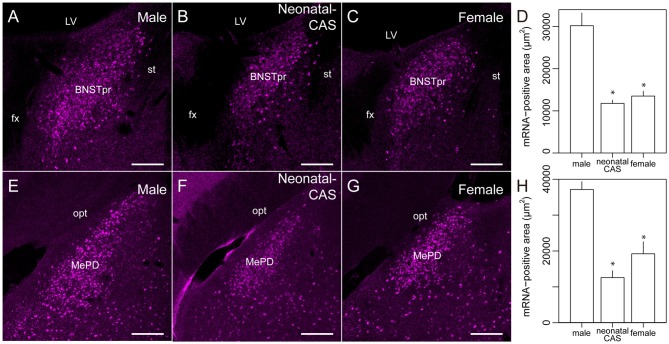
**Sexually dimorphic expression of *Moxd1* mRNA in the BNSTpr and MePD.** Representative images in the BNSTpr of intact male **(A)**, neonatally castrated male** (B)** and female **(C)** mice. **(D)** Difference in the *Moxd1* mRNA-positive area in the BNSTpr. Representative images of the MePD of male **(E)**, neonatally castrated male** (F)** and female **(G)** mice. **(H)** Difference in the *Moxd1* mRNA-positive area in the MePD. *Significant difference from intact male mice (*P* < 0.05, *n* = 4 for each, Welch’s *t*-test with Holm’s correction). BNSTpr, principal nucleus of the bed nucleus of the stria terminalis; MePD, posterodorsal part of the medial amygdala; LV, lateral ventricle; fx, fornix; st, stria terminalis; opt, optic tract. Scale bars: 200 μm.

Next, we examined the expression of *Moxd1* mRNA outside of the MPOA (Table 1). In the telencephalon, *Moxd1* mRNA was most strongly expressed and densely clustered in two regions: the BNSTpr and MePD (Table 1). Furthermore, a male-biased sex difference in the expression of *Moxd1* mRNA was recognized in the BNSTpr and MePD, and neonatal castration diminished male-dominant expressions in these areas (Figure 4). The area expressing *Moxd1* mRNA in the BNSTpr of male mice was significantly larger than that of neonatally castrated male mice and that of female mice (male: 30074 ± 3022 μm^2^, neonatally castrated male: 11764 ± 305 μm^2^, female: 13498 ± 1150 μm^2^, *n* = 4 for each, Welch’s ANOVA, *F*_(2,4.3)_ = 16.5, *P* = 0.010, *Post hoc* test, *P* < 0.05 for both, Figures 4A–D). Similar to the BNSTpr, the area expressing *Moxd1* mRNA in the MePD of male mice was significantly larger than that of the neonatally castrated male mice and that of female mice (male: 37142 ± 2195 μm^2^, neonatally castrated male: 12591 ± 1928 μm^2^, female: 19221 ± 3344 μm^2^, *n* = 4 for each, Welch’s ANOVA, *F*_(2,5.8)_ = 32.2, *P* < 0.001, *Post hoc* test, *P* < 0.05 for both, Figures 4E–H).

**Table 1:** **Density of the *Moxd1* mRNA-positive cells and its colocalization with calbindin in the telencephalon**.

Area	Mean density (Number of cells/mm^3^, *n* = 4)	Mean percentage of double-labeled cells/*Moxd1*-positive cells (*n* = 4)
**Cerebral Cortex**
Subplate (layer 6b)	2176 ± 150	0%
Tenia tecta	311 ± 62.7	77.2 ± 8.9%
Other areas (quantified in S1)	16 ± 2.3	72.4 ± 3.1%
Striatum	48 ± 17.8	3.2 ± 2.1%
Nucleus accumbens	76 ± 4.1	10.6 ± 3.0%
Hippocampus, pyramidal layer	67 ± 10.8	0%
Dentate gyrus	99 ± 15.4	0%
Septum	0	N.A.
Olfactory tubercle	79 ± 6.8	9.3 ± 1.5%
Piriform cortex	127 ± 19.4	63.9 ± 3.2%
Endopiriform nucleus	203 ± 47.9	25.0 ± 1.1%
**Amygdala**
Basolateral amygdala	195 ± 19.9	78.0 ± 6.9%
Basomedial amygdala	214 ± 25.6	71.9 ± 5.9%
Cortical amygdala	181 ± 46.4	80.0 ± 2.6%
Central amygdala	68 ± 13.0	26.2 ± 5.8%
Lateral amygdala	118 ± 11.2	49.6 ± 8.9%
Medial amygdala (posterodorsal)	>2000*	29.6 ± 4.3%
Medial amygdala (remaining part)	229 ± 23.5	37.3 ± 3.5%
**BNST**
BNST, principal nucleus	>2000*	61.4 ± 5.0%
BNST, remaining part	52 ± 6.3	27.9 ± 4.1%

*Showing apparent sex differences (also see Figure 4).

**Figure 5:**
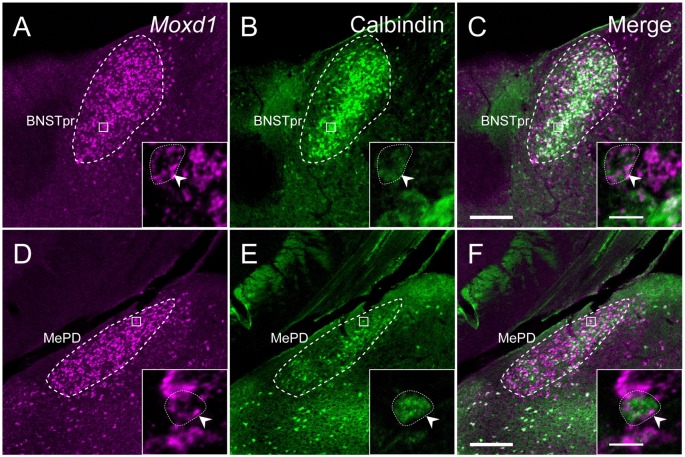
**Comparison of the distribution of *Moxd1*-positive cells with calbindin-immunoreactive cells in the BNSTpr and MePD of male mice. (A–C)** Representative images of *Moxd1* (magenta) and calbindin (green) in the BNSTpr (dashed line). **(D–F)** Representative images of *Moxd1* (magenta) and calbindin (green) in the MePD (dashed line). Scale bars: 200 μm. The inset shows a high magnification view indicated by small rectangle. Arrowhead indicates the overlap of the *Moxd1-*positive cells and the calbindin-immunoreactive cells. Scale bars: 10 μm.

**Figure 6:**
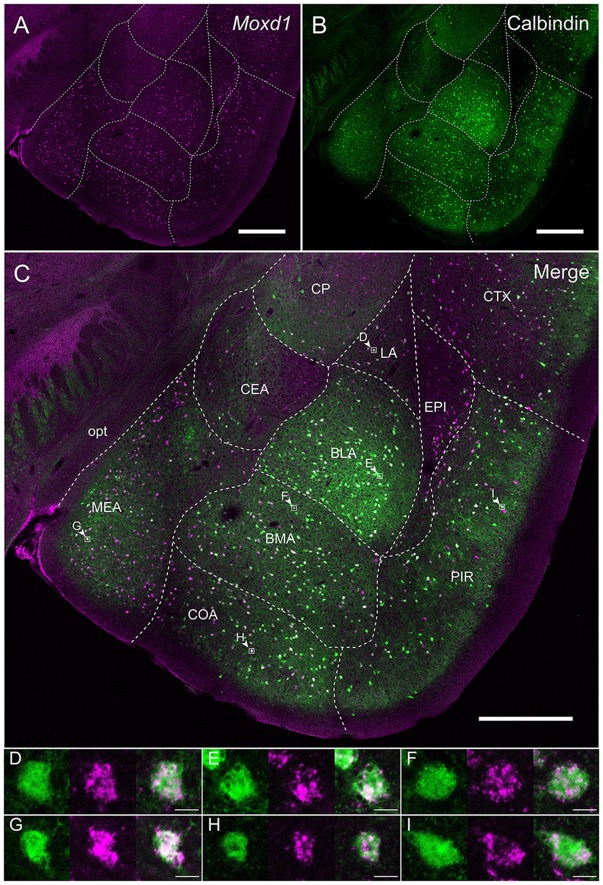
**Overlap of the *Moxd1*-positive cells and calbindin-immunoreactive cells in the amygdala nuclei and adjacent areas. (A)** *Moxd1*, **(B)** calbindin, **(C)** merged image. Note that the MePD was located in the more posterior section. BLA, basolateral amygdala; BMA, basomedial amygdala; CEA, central amygdala; COA, cortical amygdala; CP, caudoputamen; CTX, cerebral cortex; EPI, endopiriform nucleus; LA, lateral amygdala; MEA, medial amygdala; PIR, piriform cortex; opt, optic tract. Scale bars: 0.5 mm.** (D–I)** High magnification images of double-labeled cells indicated by small rectangles and arrowheads in **(C)**. **(D)** LA, **(E)** BLA, **(F)** BMA, **(G)** MEA, **(H)** COA, **(I)** PIR. Scale bars: 10 μm.

Calbindin was also strongly expressed in the BNSTpr. Double labeling of *Moxd1* mRNA and calbindin protein confirmed that the majority of the *Moxd1* mRNA-positive cells expressed calbindin in the BNSTpr (Figures 5A–C), as was observed in the SDA-POA (Figures 3A–F). The *Moxd1* mRNA-positive cells were distributed uniformly and densely in the entire BNSTpr, whereas the calbindin-ir cells were concentrated in the center area of the BNSTpr (Figures 5A–C). In the remaining part of the BNST, a moderate number of *Moxd1*-positive cells were found without any difference between sexes and immunoreactivity for calbindin (Table 1). A uniform and dense distribution of the *Moxd1*-positive cells was found in the entire MePD, whereas a small number of the calbindin-ir cells were located in the center area of the MePD (Figures 5D–F). *Moxd1* mRNA-expressing cells were found in the basolateral amygdala (BLA), basomedial amygdala (BMA), cortical amygdala (COA), piriform cortex and MEA outside of the MePD (Table 1, Figure 6). Most of the *Moxd1* mRNA-positive cells in the amygdala were calbindin-positive, except for in the central amygdala (CEA; Figure 6). There was no apparent sex difference in *Moxd1* mRNA expression outside of the MePD.

**Figure 7:**
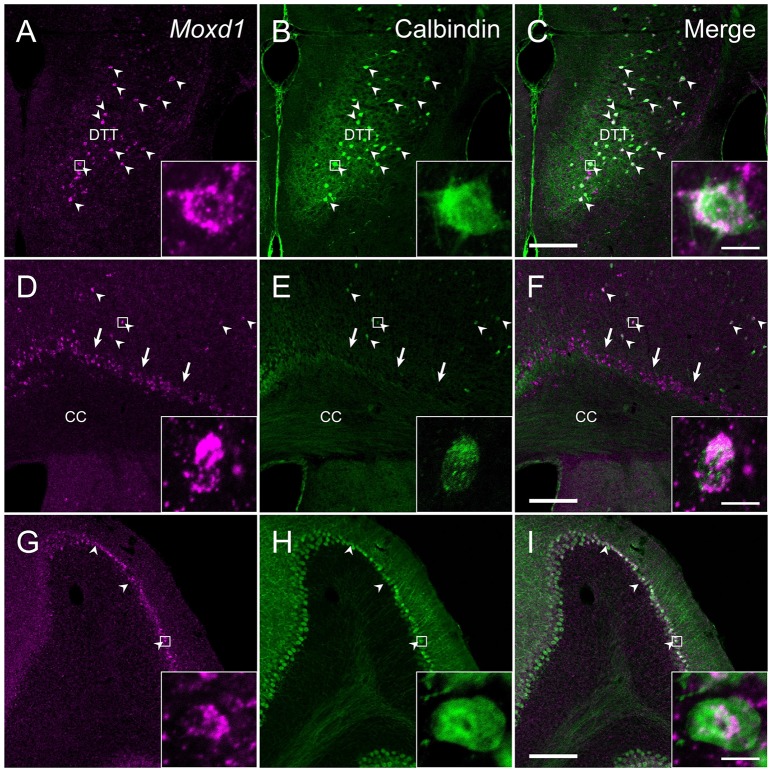
**Comparison of the distribution of *Moxd1*-positive cells with calbindin-immunoreactive cells in the dorsal tenia tecta (DTT), cerebral cortex (CTX) and cerebellum. (A–C)** Representative images of *Moxd1* (magenta) and calbindin (green) in the dorsal tenia tecta. **(D–F)** Representative images of *Moxd1* (magenta) and calbindin (green) in the CTX. **(G–I)** Representative images of *Moxd1* (magenta) and calbindin (green) in the cerebellum. Arrowhead indicates the overlap of the *Moxd1-*positive cells and the calbindin-immunoreactive cells. Arrow indicates exclusive *Moxd1* expression in the subplate layer of the CTX. Scale bars: 200 μm. Each inset shows a high magnification view of a double-labeled cell indicated by small rectangle. Scale bar: 10 μm. DTT, dorsal tenia tecta; CC, corpus callosum.

In the cerebral cortex (CTX), strong *Moxd1* signals were found in the dorsal tenia tecta (DTT) and scattered throughout the cortical layers (Figure 7A). Many of the *Moxd1* mRNA-positive cells in the DTT and cerebral cortical layer had also calbindin-ir signals (Figures 7A–F). *Moxd1* mRNA was strongly expressed in the subplate layer of the CTX as reported previously (Figure 7D; Hoerder-Suabedissen et al., 2009), whereas the *Moxd1*-positive cells in the subplate were negative for calbindin (Figures 7D–F). *Moxd1*-positive cells were scattered in the olfactory tubercle, striatum, nucleus accumbens, hippocampus and dentate gyrus, without colocalization of calbindin (Table 1).

There were few cells expressing *Moxd1* mRNA in the diencephalon and brainstem. In the cerebellar cortex, *Moxd1* mRNA-positive cells were abundantly found in Purkinje cells, where intense calbindin-ir cells were distributed (Figures 7G–I). Intense *Moxd1* signals were also found in the colloid plexus and dura.

## Discussion

The present study showed that the strong expression of *Moxd1* mRNA is limited to the SDN-POA in mice. In addition, uniform expression of *Moxd1* mRNA was observed in the BNSTpr and MePD, where sex difference has been found. The numbers of *Moxd1* mRNA-positive cells in the SDN-POA, BNSTpr and MePD in male mice was greater than that in female mice. Neonatal castration resulted in a reduced number of *Moxd1* mRNA-positive cells in the SDN-POA of adult male mice, whereas the castration of adult males did not change the number of *Moxd1* mRNA-positive cells in the SDN-POA, suggesting that the expression of *Moxd1* mRNA in the SDN-POA is determined by the hormonal milieu during the perinatal period and that the expression is independent of the activation effect of gonadal steroids in adulthood. *Moxd1* expression in the MPOA was more restricted to the SDN-POA than expression of calbindin. Collectively, these data suggest that *Moxd1* serves as a useful molecular marker of sexually dimorphic nuclei in mice.

### Neural Circuitry Containing Neurons that Strongly Express *Moxd1*

*Moxd1* is abundantly expressed in the SDN-POA, BNSTpr and MePD in a sexually dimorphic manner, where the cell number and volume show a male-biased sex difference which is determined by high testosterone level during the perinatal period (Gilmore et al., 2012; Campi et al., 2013; Moe et al., 2016a). Among these nuclei, BNSTpr and MePD are included in the medial extended amygdala, which serve male reproductive circuits via pheromonal inputs from accessary olfactory bulb and contain various sexually dimorphic structures (Newman, 1999; de Olmos and Heimer, 1999; Martínez-García et al., 2012). Neurocircuit, neurochemical and functional similarity were found between BNSTpr and MePD neurons in relation to the SDN-POA. The SDN-POA has some efferent and afferent connections to the BNSTpr and MePD (Simerly and Swanson, 1988; Maejima et al., 2015). MePD neurons send their axons most abundantly to the BNSTpr (Dong et al., 2001; Been and Petrulis, 2011). The MePD receives dense input from the vomeronasal organ via anterior and posterior accessory olfactory bulb to detect pheromones (Von Campenhausen and Mori, 2000; Mohedano-Moriano et al., 2007; Cádiz-Moretti et al., 2016), and the BNSTpr also receives inputs from anterior accessory olfactory bulb (Mohedano-Moriano et al., 2007, 2008), suggesting that odor/pheromones regulate the MePD neurons and BNSTpr, and subsequently SDN-POA neurons. In fact, female odor has been shown to induce Fos immunoreactivity in MePD neurons that project to the BNST and MPOA (Been and Petrulis, 2011). Male sexual behavior toward females induces fos expression in the MePD, BNSTpr and SDA-POA (Heeb and Yahr, 1996; Coolen et al., 1998). Rich expression of gonadal steroid receptors in these nuclei implies their role in sexual behaviors which are under the influence of gonadal steroids (Shughrue et al., 1997).

### *Moxd1* Is a Better Marker for Sexually Dimorphic Nuclei than Calbindin

The SDN-POA was larger in volume in male than in female mammals (Gorski et al., 1978; a list in Campi et al., 2013). However, the sexual dimorphism of the SDN-POA in laboratory mice was not recognized or was very subtle in Nissl-stained sections (Young, 1982; Brown et al., 1999). Recently, calbindin has been used to visualize the SDN-POA in mice as well as rats, musk shrews and common marmosets in a sexually dimorphic pattern (Sickel and McCarthy, 2000; Edelmann et al., 2007; Bodo and Rissman, 2008; Orikasa and Sakuma, 2010; Jahan et al., 2015; Moe et al., 2016a,b). The sexual dimorphism of calbindin-ir cells in the MPOA depends on the presence of testosterone during the neonatal period and is independent of the gonadal steroid hormone level in adult animals (Sickel and McCarthy, 2000; Orikasa and Sakuma, 2010). Sexually dimorphic markers for the rat MPOA, such as the androgen receptor (McAbee and DonCarlos, 1998) and genes that are expressed in the SDN-POA-equivalent region (Simerly et al., 1986), have failed to show sexual dimorphism in mice (Jahan et al., 2015). So far, calbindin has been the only marker for the mouse SDN-POA.

However, calbindin-ir cells are not restricted to the SDN-POA and BNSTpr but exist abundantly in the MPOA, BNST and MEA outside of the SDN-POA, BNSTpr and MePD, respectively. Moreover, we found that the majority of *Trh*- and oxytocin-positive neurons in the MPOA also expressed calbindin (Figure 3), consistent with the rat MPOA (Arai et al., 1994). Therefore, optogenetic and pharmacogenetic studies using a Cre-driver mouse targeting calbindin may affect a variety of neurons surrounding the sexually dimorphic nuclei.

Unlike calbindin, *Moxd1* is expressed more specific to sexually dimorphic nuclei and more uniformly expressed in the SDN-POA, BNSTpr and MePD. The majority of the *Moxd1*-positive cells in sexually dimorphic nuclei were calbindin-ir and not positive for *Trh* or oxytocin (Figure 3). Thus, together combined with a local injection of adeno-associated viral vector, the *Moxd1* gene may enable us to manipulate neurons in sexually dimorphic nuclei to examine the effect on sexual behaviors and to elucidate the afferent and efferent connections of sexually dimorphic neurons (Schwarz et al., 2015; Lerner et al., 2016). *Moxd1* encodes a monooxygenase, DBH-like 1, that is localized in the endoplasmic reticulum and predicted to hydroxylate a hydrophobic substrate based on its amino acid sequence similar to dopamine β-hydroxylase (Xin et al., 2004). Because the substrates of Moxd1 have not been identified, the biological roles of Moxd1 in sexually dimorphic neurons are not known. A frequent colocalization of *Moxd1* and calbindin in sexually dimorphic nuclei implies that Moxd1 and calbindin work together to regulate intracellular calcium released from the smooth endoplasmic reticulum. Future studies to identify a regulatory network inducing the *Moxd1* gene expression may elucidate the mechanism underlying the development of sexual dimorphic nuclei.

## Author Contributions

YT, ST and HF conceived and designed the experiments. YT, SY, SO and MK performed the experiments. YT analyzed the data. YT and KT contributed reagents/materials/analysis tools. YT, ST and HF wrote the article. All authors approved this article to be published and agree to be accountable for the content of the work.

## Funding

This work was supported by The Japan Health Foundation (to YT), JSPS KAKENHI (Grant Number 15K18364 to YT; 26220207 to HF; 16K15187 to HF; 26507003 to HF) and Ministry of Education, Culture, Sports, Science and Technology (MEXT) KAKENHI (Grant Number 15H05935 to HF).

## Conflict of Interest Statement

The authors declare that the research was conducted in the absence of any commercial or financial relationships that could be construed as a potential conflict of interest.

## Footnotes

^1^http://mouse.brain-map.org/agea

## Abbreviations

3v: Third ventricle
ACN: Anterior commissural nucleus
BLA: Basolateral amygdala
BMA: Basomedial amygdala
BNST: Bed nucleus of the stria terminalis
BNSTpr: Principal nucleus of the bed nucleus of the stria terminalis
CAS: castrated
CC: Corpus callosum
CEA: Central amygdala
COA: Cortical amygdala
CP: Caudoputamen
CTX: Cerebral cortex
DTT: Dorsal tenia tecta
EPI: Endopiriform nucleus
fx: Fornix
LA: Lateral amygdala
LV: Lateral ventricle
MEA: Medial amygdala
MePD: Medial amygdala, posterodorsal
MPN: Medial preoptic nucleus
MPNm: Medial preoptic nucleus, medial part
MPOA: Medial preoptic area
opt: Optic tract
OVX: ovariectomize
PIR: Piriform cortex
SDN-POA: Sexually dimorphic nucleus of the preoptic area
st: Stria terminalis
vMPOA: Ventral part of the medial preoptic area.
